# NMR-Based Metabolomics for Geographical Discrimination of *Adhatoda vasica* Leaves

**DOI:** 10.3390/plants12030453

**Published:** 2023-01-18

**Authors:** Muhammad Jahangir, Atta-ur-Rehman, Ibrahim Bayoumi Abdel Farid, Robert Verpoorte, Imran Khan, Jiangnan Peng

**Affiliations:** 1 Department of Food Science & Technology, The University of Haripur, Haripur 22620, Pakistan; 2 Khyber Pakhtunkwa Food Safety & Halal Food Authority, Peshawar 25110, Pakistan; 3 Biology Department, College of Science, Jouf University, Sakaka P.O. Box 2014, Saudi Arabia; 4 Botany Department, Faculty of Science, Aswan University, Aswan 81528, Egypt; 5 Natural Products Laboratory, Institute of Biology, Leiden University, 2300 RA Leiden, The Netherlands; 6 Department of Chemistry, School of Computer, Mathematical & Natural Sciences, Morgan State University, Baltimore, MD 21251, USA

**Keywords:** *Adhatoda vasica*, geographical variation, herbal medicine, ^1^H-NMR spectroscopy, metabolomics

## Abstract

*Adhatoda vasica* (L.), Nees is a widespread plant in Asia. It is used in Ayurvedic and Unani medications for the management of various infections and health disorders, especially as a decoction to treat cough, chronic bronchitis, and asthma. Although it has a diverse metabolomic profile, this plant is particularly known for its alkaloids. The present study is the first to report a broad range of present compounds, e.g., α-linolenic acid, acetate, alanine, threonine, valine, glutamate, malate, fumaric acid, sucrose, β-glucose, kaempferol analogues, quercetin analogues, luteolin, flavone glucoside, vasicine and vasicinone, which were identified by NMR spectroscopy-based metabolomics. Multivariate data analysis was used to analyze ^1^H-NMR bucketed data from a number of *Adhatoda vasica* leave samples collected from eight different regions in Pakistan. The results showed large variability in metabolomic fingerprints. The major difference was on the basis of longitude/latitude and altitude of the areas, with both primary and secondary metabolites discriminating the samples from various regions.

## 1. Introduction

*Adhatoda vasica* (L.), Nees is a well-known medicinal plant in Ayurvedic and Unani medicine. It has been used for the treatment of various disorders, such as for respiratory tract ailments [1], radiomodulation [2], hypoglycaemic, cardiovascular prevention, antituberculosis, antiviral, hepatoprotective, antimutagenic and antioxidant [3]. The frequent use of *A. vasica* has resulted in its inclusion in the WHO manual “*The Use of Traditional Medicine in Primary Health Care*” which is intended for health workers in Southeast Asia to keep them informed on the utility of their surrounding flora; in the case of *A. vasica*, this concerns the treatment of cough, asthma and bleeding piles. This plant can be used both for adults and children for prolonged periods [4]. The Vasaka plant is known for pyrroquinazoline alkaloids, such as vasicine, vasicol and vasicinone. Vasicine, the major alkaloid, was shown to have acetylcholinesterase inhibitory activity [5]. 

The herbal medicine industry uses decoction to extract *A. vasica* leaves. This single plant extract contains hundreds of organic chemicals, and it will be difficult to characterize a single active ingredients from the complex decoction mixture [6]; even the occurrence of several compounds may interact for having the phytotherapeutic effect. Herbal medicines are sourced from a number different geographical locations, which means that they may have qualitative and quantitative differences in the spectra of compounds present in the plant, i.e., in the metabolome [7,8]. The identification of the active ingredient(s) and knowing their variability in the herbal material is a major challenge for quality control to ensure reproducible herbal medicines [6]. 

Multiple chemometrics techniques have been reported for the characterization of bioactive constituents of medicinal plants by using various analytical instruments, such as NMR, GC-MS, HPLC, etc. However, NMR has the advantage of a non-destructive, highly reproducible and robust method for both qualitative and quantitative analysis of biological materials, with a simple sample preparation and ease of analysis. It has successfully been used for metabolic fingerprinting of various herbal materials [9]. The ^1^H-NMR-based metabolic evaluation has also been used for quality control of herbal materials [10,11,12]. As a chemometric approach, the analyzed data are further evaluated through multivariate data analysis, including principal component analysis, to extract information out of the large amount of data obtained in metabolomics studies. This allows the identification of discriminating compounds in various samples, i.e., to identify the source of the material or identify the compounds related to pharmacological activity or plant resistance against pests and diseases [13,14,15,16]. The present work is a first step in developing such a metabolomics method for the quality control of *A. vasica* leaves for the use as phytomedicine. This includes the characterization of its metabolites, through ^1^H-NMR-based metabolomics. Plant material was collected in different regions and analyzed by NMR-based metabolomics. Principal component analysis and hierarchical clustering were used to achieve insight in the variability of the chemistry of the leaves and the effect of altitude under the plants collection conditions.

## 2. Results and Discussion

The leaves of *A. vasica* plants from eight different regions (Table 1) namely: Abbottabad, Havelian, Muzafarabad, Khanpur, Taxila, Haripur, Ghazi and Rawalpindi) of Pakistan were collected by considering the altitude as an important factor for sample collection, and numbered accordingly. 

**Figure 1:**
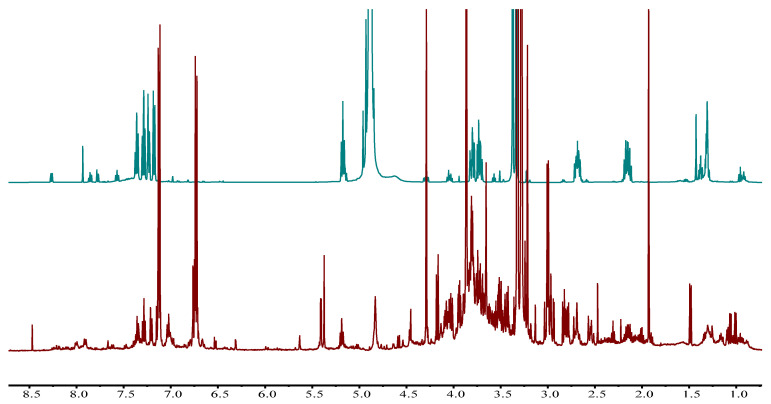
^1^H-NMR spectrum (500 MHz) of alkaloid fraction (blue color) superimposed on ^1^H-NMR spectrum of mixture extract (brown color) from the sample collected at Khanpur (location 4 as per Table 1) both in 50% CD_3_OD–D_2_O.

A total of sixteen compounds were identified: α-linolenic acid, acetate, alanine, threonine, valine, glutamate, malate, fumarate, sucrose, β-glucose, kaempferol analogues, quercetin analogues, luteolin, flavone glucoside, vasicine and vasicinone (Table 2). All these compounds were confirmed through 1D and 2D NMR spectral analysis and comparison with the previously reported data as well. Vasicine and vasicinone are the most important known bioactive compounds of this plant, and they could be detected and identified in the ^1^H-NMR spectrum of the crude extract by overlay the ^1^H-NMR of the purified alkaloid fraction (Figure 1) and by comparison with previously reported data of the pure alkaloids from *A. vasica* [17]. 

The NMR-based metabolomics coupled with multivariate data analysis has proved its strength for quality control that was limited to detection and/or quantification of targeted compounds [18]; however, the methodology as followed here has previously proved the similar results of chemometric methods and official conventional methods for the targeted compounds as well, and so no further separation is suggested [19,20].

**Figure 2:**
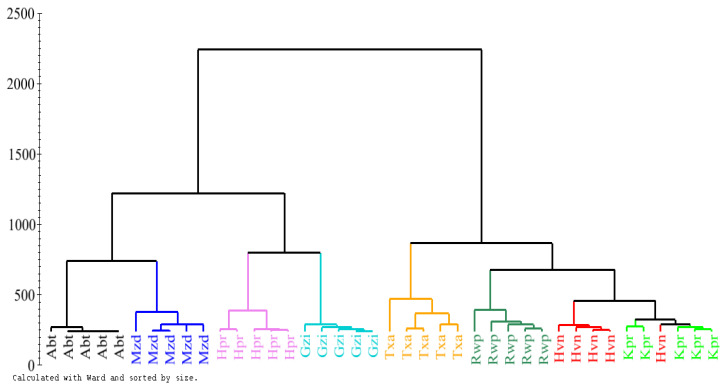
Dendrogram constructed on ^1^H-NMR of samples of eight regions (five replications each) of *Adhatoda vasica* leaves from various regions, i.e., Abbottabad (Abt) at altitude of 4005.91 ft, Muzaffarabad (Mzd) at altitude of 2444.23 ft, Haripur (Hpr) at altitude of 1578.08 ft, Gazi (Gzi) at altitude of 1236.88 ft, Taxila (Txa) at altitude of 1784.78 ft, Rawalpindi (Rwp) at altitude of 1558.40 ft, Havelian (Hvn) at altitude of 2808.4 ft and Khanpur (Kpr) at altitude of 1975.07 ft.

**Figure 3:**
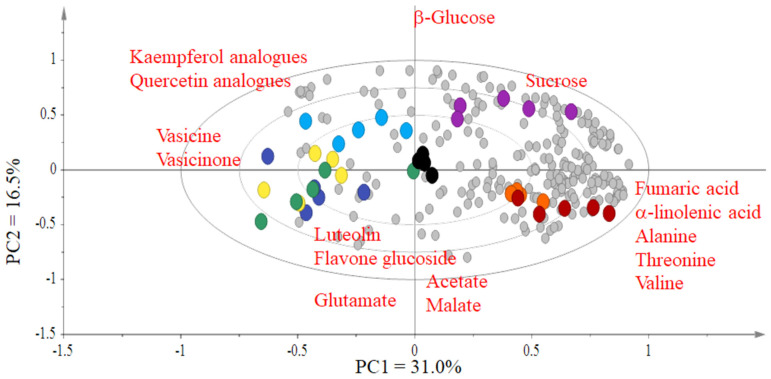
Biplot of Principal Component Analysis (PCA) (R2/Q2 = 0.787/0.541) for the NMR spectral data of the samples from the different regions: *Adhatoda vasica* leaves from various regions (Abbottabad ●, Muzaffarabad ●, Haripur ●, Gazi ●, Taxila ●, Rawalpindi ●, Havelian ●, Khanpur ●, NMR shifts of metabolites ●.)

**Figure 4:**
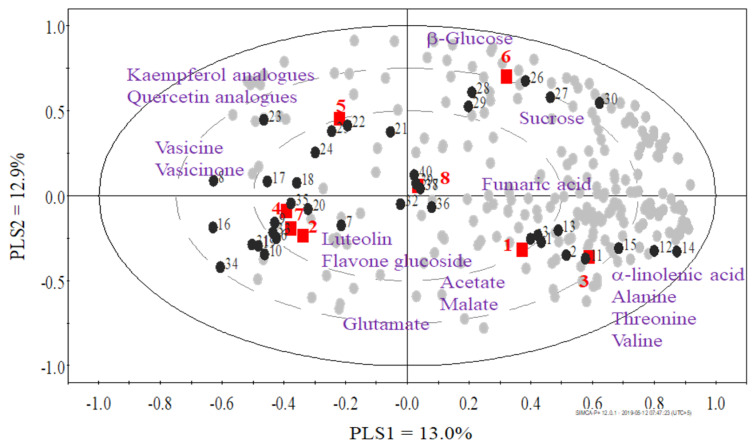
Principal Least Square Discriminant Analysis (PLS-DA) (R2/Q2 = 0.356/0.003) of the NMR spectral data of *Adhatoda vasica* leaves. The grouping is based on different regions (i.e., Gazi = 1◼, Haripur = 2◼, Taxila = 3◼, Khanpur = 4◼, Rawaplindi = 5◼, Havelian = 6◼, Abbottabad = 7◼ and Muzaffarabad = 8◼) and numbering is based on latitude.

The ^1^H-NMR data were bucketed and analyzed by various multivariate data analysis methods: hierarchical cluster analysis (Figure 2), PCA (Figure 3) and PLS-DA (Figure 4). The cluster analysis clearly grouped all regions, proving a clear discrimination of all samples, but the series of samples from various regions was not in the sequence of their altitude (Table 1), which proves that this factor alone does not fully explain the clustering of plant samples. When various regions were aligned on the basis of longitude and latitude (the samples grouped on the basis of their region, especially longitude (Figure 2, Table 2), we could draw a pattern in alterations up to metabolite level, accordingly (Figure 2, Figure 3 and Figure 4). Similarly, the altitude of regions did not show a more defined relationship in our findings; however, it revealed an impact. 

Here we report that the metabolomic composition of all samples from various regions is qualitatively similar but have clear discrimination in PCA (Figure 3) and PLS-DA (Figure 4) that reveals the quantitative differences among metabolites, including the characteristic alkaloids of *A. vasica*. As in industrial applications, the plant material is collected from various geographic locations, producing the similar total yield industrial applications, but the biologically active ingredients matter the most, so these differences, especially in case of bioactive secondary metabolites, i.e., the lower amount of alkaloids in case of *A. vasica*, impart negative impact on the nutraceutical quality of a medicinal plant, which is a major concern for their use as medicine [21], while the primary metabolites, i.e., sugars and others, may also impart an impact on taste and other characteristics.

Studying the outcomes in detail reveals that the *A. vasica* samples from Muzaffarabad (3) and Abbottabad (1) regions are grouped closely together in hierarchical clustering (Figure 2). This is also evident in principal component analysis (Figure 3) and partial least squares discriminant analysis (Figure 4). Discrimination from other samples is due to the signals of α-linolenic acid, alanine, threonine and valine. It is evident (Figure 2, Figure 3 and Figure 4) that the samples from Khanpur (4), Havalian (6) and Rawalpindi (5) were aligned with each other and clearly discriminated from others due to the signals of luteolin, flavone glucoside, vasicine and vasicinone.

Although the samples collected from Taxila (3) were identified similar to those collected from Khanpur (4), Rawalpindi (5) and Havelian (6), as in hierarchical clustering (Figure 2), but these kept their identity within the same group by discriminating itself in PLS-DA (Figure 4) having higher signals for Kaempferol analogues and quercetin analogues. As aforementioned, the samples collected from Khanpur (4), Rawalpindi (5) and Havelian (6) identified for luteolin and flavone glucoside as discriminated metabolites. However, in the case of alkaloids, i.e., vasicine and vasicinone, it appears to be the discriminating factor for the plants collected from aforementioned areas, and so the plants from all these areas appear in similar groups as observed in hierarchical clustering (Figure 2).

Two locations, i.e., Haripur (2) and Gazi (1), were grouped near each other and were discriminated from others due to higher signals of β-glucose, sucrose, and fumaric acid. Longitude and altitude are contributing factors to define the environment of a region, maybe because of light (intensity, day length, UV) and temperature [22]. Besides this, it is evident that a difference in geology in geographical regions also imparts the metabolome, resulting in changes in the bioactivity of plants from the same species of plants [8]. In our results, the pattern of metabolomics discrimination is identified on the base of longitude, but it does not mean that it is the only variable impeding the biological processes in plants. It is well documented that latitude is an important factor, such as in the case of a controlled experiment on *Arabidopsis thaliana*; a variation in plant size and relative growth rate (RGR) was observed along a latitudinal gradient. the plants at high latitudes are reported to have a smaller size for their seeds, cotyledon width and leaf area as compared to those from low latitudes [23]. Nevertheless, as it is the combination of altitude, latitude and longitude that effects the weather of a point on earth and so imparts metabolomic alteration in plants [24,25]. Unfortunately, the longitude of a position received less attention for biochemical alterations in plant materials; that is also important for specific positioning of a plant material. 

Bioactive compounds occurring in plant material are part of a multi-component mixture, so their isolation, identification and structure elucidation remains a challenge [26]. Changes in such a complex matrix (including undesirable compounds) may alter the bioavailability and bio-efficacy of the active ingredients, e.g., through synergy [27]. Decoctions are used mainly as an extraction method for herbs in industry, so besides quantity of the desired active compound(s), the extraction efficiency of a diverse range of metabolites is also an important quality trait [28]. For example, the intracellular metabolite extraction efficiency was found to be dependent on the extraction method [29]. Based on our findings, it is recommended to use metabolomic analysis coupled with multivariate data analysis to define the plant material, i.e., the characteristics of the required plant material for an optimal reproducible pharmaceutical material and to describe protocols for the agricultural production and the subsequent extraction procedure to obtain this final product. Eventually the desired quality might be obtained by mixing different batches to arrive at the defined quality.

## 3. Material and Methods

### 3.1. Collection of Plant Material

The plant leaves (fresh-looking top few leaves that were free from herbivory/infections and facing the sun side) were collected in the month of March from various areas, namely, Abbottabad (34°11′59.6″ N 73°14′47.0″ E) at 4005.91 ft, Havelian (34°03′06.9″ N 73°09′26.5″ E) at 2808.4 ft., Muzafarabad (34°20′56.0″ N 73°29′19.3″ E) at 2444.23 ft., Khanpur (33°48′55.0″ N 72°55′16.8″ E) at 1975.07 ft., Taxila (33°46′00.3″ N 72°52′20.1″ E) at 1784.78 ft., Haripur (33°59′33.7″ N 72°50′51.5″ E) at 1578.08 ft., Rawalpindi (33°31′49.4″ N 73°03′00.7″ E) at 1558.40 ft. and Ghazi (34°01′29.6″ N 72°40′49.6″ E) at 1236. 88 ft., The altitude was measured through https://www.daftlogic.com/sandbox-google-maps-find-altitude.htm (accessed on 20 March 2022), whereas the latitude & longitude was measured with Google maps. A total of eight (8) different locations were identified based on the natural availability of plant material, where five (5) replications (each from different plant) from each location were collected. The botanical authentication was conducted at the Department of Botany, University of Malakand, Chakdara, Pakistan, and a voucher specimen (UOM/BGH/20/109) was deposited in the herbarium.

### 3.2. Drying of Leaves

A total of 50 g sample (leaves) for each replication was collected and dried in the shade for three days before further freezedried and stored in 50 mL polypropylene centrifuge tubes, until further used for extraction and/or NMR analysis.

### 3.3. Extraction of Leaves for Mixture Analysis and NMR Measurements

All of the solvents and reagents were purchased from Sigma-Aldrich, Germany. Extraction of freeze-dried plant material by using 50% methanol-*d*_4_ in D_2_O (KH_2_PO_4_ buffer, pH 6.0) containing 0.05% TSP (trimethyl silyl propionic acid sodium salt, *w*/*v*) and NMR measurements was carried out by using a 500 MHz Bruker DMX-500 NMR spectrometer (Bruker, Karlsruhe, Germany) operating at a proton NMR frequency of 500.13 MHz, with the same protocol as reported previously [18]. Compounds were identified by 1D (Figure 1) and 2D (Figures S1–S3; Supplementary Data) NMR spectral analysis and confirmed by comparison with the previously reported data as well [30,31].

### 3.4. Extraction Alkaloids

All the solvents and reagents were purchased from Sigma-Aldrich, Germany. Extraction and isolation of alkaloid fraction was carried out as reported previously through liquid-liquid (acid/base) extraction followed by preparatory TLC for extraction of crude alkaloid fraction [32]. The freeze-dried ground leaves (1 kg) were extracted with ethanol (3 times with 3 L, each times), and the extract was evaporated to dryness that yielded a gummy green material which was further extracted with hot, double deionized water (3 times with 500 mL, each times), cooled and filtered; where the chlorophyll part was discarded, the aqueous solution was further extracted with chloroform, (3 times with 250 mL, each times). The aqueous layer was basified with 5% NaOH (pH 8–9) and further extracted with chloroform (CHCI_3_) (3 times with 250 mL, each time). Furthermore, this chloroform layer was extracted with 5% HCI (3 times with 200 mL, each time), and the acidic solution was basified with NaOH and extracted once again with chloroform until the organic layer was free of alkaloids. The alkaloid fraction was separated gradually, dried, confirmed through Dragendorffs reagent and further analyzed by NMR to clearly identify various alkaloids in it, whereas this data set was also used further to support our findings in mixture analysis by NMR.

### 3.5. Computational Processing of NMR Data

The Mnova (v. 6.2.1-7569, Mesterlab Research S.L., Santiago de Compostela, Spain) software was used for ^1^H-NMR and 2D NMR spectral analysis of the alkaloid fraction and the total leave extract. Results of previous reports on this plant were referenced for identification of metabolites [18,33]. Superimposing the NMR spectrum of the alkaloid fraction (as identified and mentioned above) on the leave extract spectrum was used to confirm the identification of the alkaloids in the crude leave extract.

As second step, the bucketing of the one-dimensional proton NMR spectrum was performed by the AMIX software (Bruker), where the spectrum was normalized manually with solvent peak (CD_3_OD) by hundred and binned to equal width (0.04) throughout the spectral width of δ 0.3–10.0, followed by transforming the bucketed files to an ASCII file. The scaling was conducted by total intensity, whereas regions of δ 4.75–4.9 and δ 3.28–3.34 were excluded from the analysis because of the residual signal of solvents [18,34]. The hierarchical clustering analysis principal component analysis (PCA) and partial least square-discriminant analysis (PLS-DA) were performed with the SIMCA-P software (v. 11.0, Umetrics, Umea, Sweden) by using unit variance method, respectively [13,35].

## 4. Conclusions

Our results are evident for the geographic alteration in the metabolomic profile of the studied plant material. The NMR-based analysis as non-targeted analysis of plant material, coupled with multivariate data analysis, effectively described the sample variability and clustering corresponding to metabolites contribution as specific to geographic region. The plants from Taxila (3), Khanpur (4), Rawalpindi (5) and Havelian (6) regions are discriminated due to the ingredients of interest from the *A. vasica*, especially the characteristic alkaloids of this plant, i.e., vasicine and vasicinone. 

However, the plants collected from Taxila (3) were discriminated by kaempferol analogues and quercetin analogues from those of Khanpur (4), Rawalpindi (5) and Havelian (6) that were discriminated by luteolin and flavone glucoside. However, plants from all other regions were more or less discriminated by amino acids, organic acids, sugars, etc., in generic terms.

Geographic positioning of plant growth matters a lot, and plant materials can be tracked for their geographic positioning as well as bioactivity potential in industry through a metabolomic approach. This report proves that NMR metabolomic profiling coupled with multivariate data analysis has a great potential to be used for quality control of herbs, such as in this case of *Adhatoda vasica*. This also demonstrates that care and proper analysis must be conducted by herbal processors while importing herbs from different regions or countries.

## Figures and Tables

**Table 1:** Regions sorted by the altitude and longitude.

S.N	Name	Altitude	Latitude	Longitude
1	Abbottabad	4005.91 ft.	34°11′59.6″ N	73°14′47.0″ E
2	Havelian	2808.4 ft.	34°03′06.9″ N	73°09′26.5″ E
3	Muzafarabad	2444.23 ft.	34°20′56.0″ N	73°29′19.3″ E
4	Khanpur	1975.07 ft.	33°48′55.0″ N	72°55′16.8″ E
5	Taxila	1784.78 ft.	33°46′00.3″ N	72°52′20.1″ E
6	Haripur	1578.08 ft.	33°59′33.7″ N	72°50′51.5″ E
7	Rawalpindi	1558.40 ft.	33°31′49.4″ N	73°03′00.7″ E
8	Ghazi	1236.88 ft.	34°01′29.6″ N	72°40′49.6″ E

**Table 2:** ^1^H chemical shifts (δ) and coupling constants (Hz) of metabolite of Vasaka leaves obtained using 1D and 2D NMR experiments (CD_3_OD-KH_2_PO_4_ in D_2_O (pH 6.0).

	Metabolite	Chemical (δ) Shift and Coupling Constant (*J*, Hz)
1.	α-linolenic acid	δ 0.98 (t, *J* = 7.5)
2.	Acetate	δ 1.94 (s)
3.	Alanine	δ 1.49 (d, *J* = 7.4), δ 3.72 (d, *J* = 7.5)
4.	Threonine	δ 1.33 (d, *J* = 6.6), δ 4.23 (m),
5.	Valine	δ 1.01 (d, *J* = 7), δ 1.06 (d, *J* = 7), δ 2.28 (m), δ 3.54 (d, *J* = 16)
6.	Glutamate	δ 2.14 (m), δ 2.68 (m), δ 3.79 (dd, *J* = 7.8, 1.4)
7.	Malate	δ 2.82 (dd, *J* = 17.0, 8.5), δ 2.95 (dd, *J* = 16.8, 4.0), δ 3.94 (dd, *J* = 8.3, 4.2),
8.	Fumaric acid	δ 6.53 (s)
9.	Sucrose	δ 3.47 (dd, *J* = 9.9, 3.8), δ 3.51 (dd, *J* = 9.9, 4.0), δ 5.4 (d, *J* = 4), δ 4.17 (d, *J* = 8.9)
10.	β-Glucose	δ 4.58 (d, *J* =8)
11.	Kaempferol analogues	δ 6.67 (d, *J* = 2.4), δ 6.77 (d, *J* = 2.4), δ 7.03 (d, *J* = 8.8), δ 8.00 (d, *J* = 8.8)
12.	Quercetin analogues	δ 6.31 (d, *J* = 2.0), δ 6.52 (d, *J* = 2.0), δ 6.98 (d, *J* = 8.8), δ 7.61 (dd, 2.2, 8.8), δ 7.68 (d, *J* = 2.2)
13.	Luteolin	δ 6.32 (*J* = 2.0), δ 6.52 (*J* = 2.0), δ 6.67 (*J* = 2.6), δ 6.73 (d, *J* = 8.5), δ 6.72 (s), δ 6.8 (dd, *J* = 2.6, 8.5),
14.	Flavone glucoside	δ 6.42 (s), δ 6.79 (s), δ 7.05 (d, *J* = 8.9), δ 7.91 (d, *J* = 8.9),
15.	Vasicine	δ 2.14 (m), δ 2.70 (m), δ 3.68 (dd, *J* = 2.4, *J* = 12.8), δ 3.76 (m), δ 4.29 (s), δ 5.18 (t, *J* = 2.0), δ 6.73 (d, *J* = 9.0), δ 7.13 (d, *J* = 8.9), δ 7.36 (m)
16.	Vasicinone	δ 2.17 (m), δ 2.66 (m), δ 5.19 (t), δ 4.00 (m), δ 4.26 (m), δ 7.64 (dd, *J* = 2.48, *J* = 8.84), δ 7.91 (m), δ 8.25 (d, *J* = 7.6).

## Data Availability

Not Applicable.

## Supplementary Materials

The following supporting information can be downloaded at: https://www.mdpi.com/article/10.3390/plants12030453/s1, Figure S1: Supplementary data for J-Res spectra of Adhatoda Vasica leaf extract; Figure S2: Supplementary data for 1H–1H COSY spectra of Adhatoda Vasica leaf extract; Figure S3: Supplementary data for 1HMBC spectra of Adhatoda Vasica leaf extract.
