# Supplementary material for: NMR-Based Metabolomics for Geographical Discrimination of Adhatoda vasica Leaves
Source: Plants (Basel). 2023 Jan 18;12(3):453. doi: 10.3390/plants12030453 (PMC9921906; doi:10.3390/plants12030453)
Supplement: Supplementary file 1 [file plants-12-00453-s001.zip › plants-2062281-Supplementary.pdf]

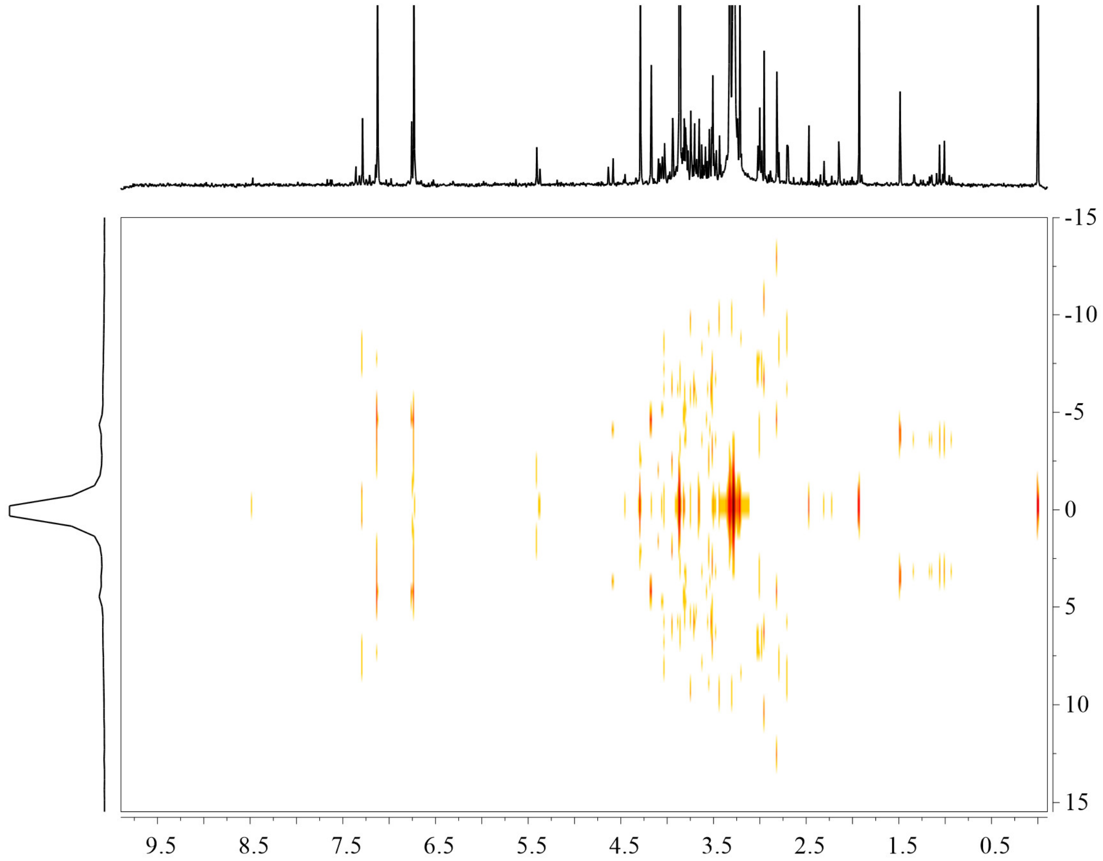

Figure S1. Supplementary data for *J*-Res spectra of *Adhatoda Vasica* leaf extract.

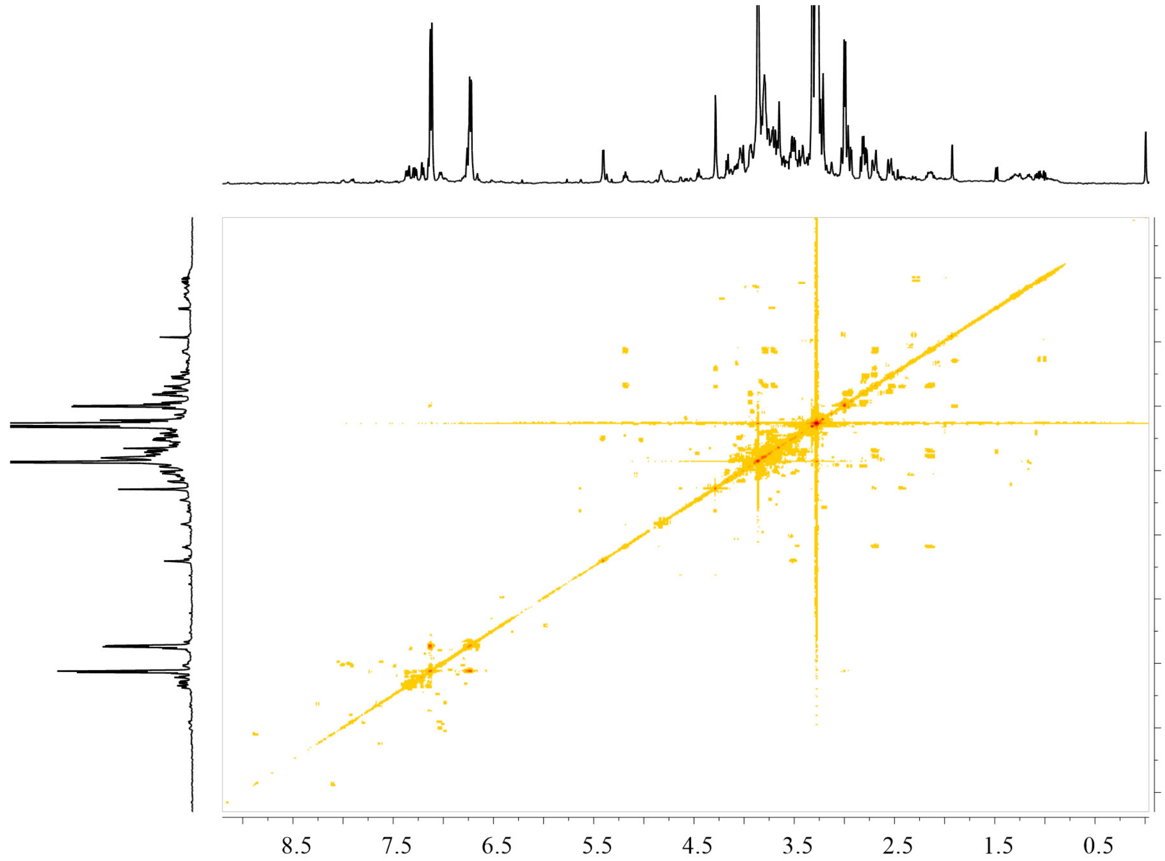

Figure S2. Supplementary data for $^{1}H - ^{1}H$ *COSY* spectra of *Adhatoda Vasica* leaf extract.

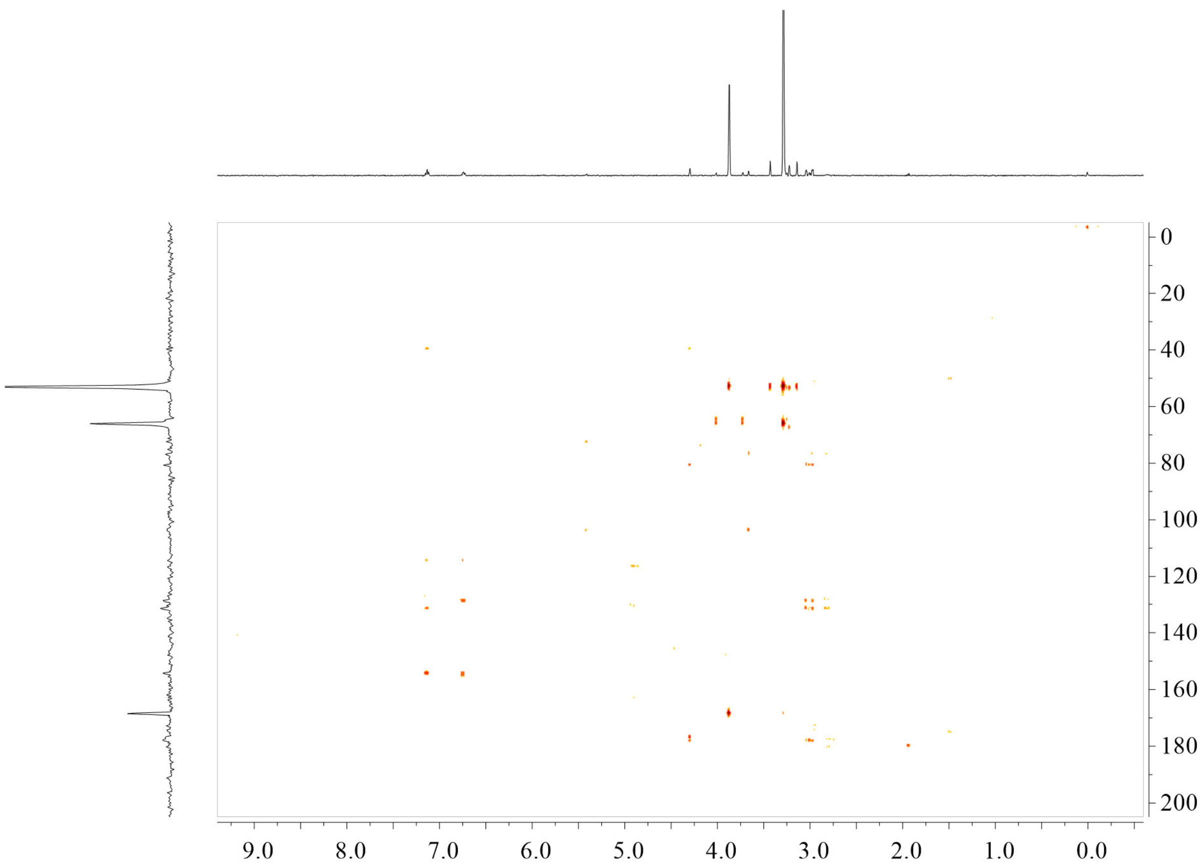

Figure S3. Supplementary data for [1]HMBC spectra of *Adhatoda Vasica* leaf extract.
